# *Cucujus tulliae* sp. n. – an endemic Mediterranean saproxylic beetle from genus *Cucujus* Fabricius, 1775 (Coleoptera, Cucujidae), and keys for identification of adults and larvae native to Europe

**DOI:** 10.3897/zookeys.212.3254

**Published:** 2012-07-30

**Authors:** Teresa Bonacci, Antonio Mazzei, Jakub Horák, Pietro Brandmayr

**Affiliations:** 1 Dipartimento di Ecologia, Università della Calabria, via P. Bucci, cubo 4b, 87036 Rende (CS), Italy; 2 Czech University of Life Sciences, Faculty of Forestry and Wood Sciences, Kamýcká 1176, CZ–16521, Prague 6, Czech Republic; 3 Silva Tarouca Research Institute for Landscape and Ornamental Gardening, Květnové náměstí 391, CZ–252 43 Průhonice, Czech Republic

**Keywords:** Calabria, *Cucujus cinnaberinus*, *Cucujus haematodes caucasicus*, Italy, Sila National Park, old–growth forests, *Pinus laricio*, relict species, larval taxonomy

## Abstract

*Cucujus tulliae* **sp. n.** is described as a new member of genus *Cucujus* Fabricius, 1775 (Coleoptera, Cucujidae), which enumerates at present eleven species distributed in Eurasia and northern America. This saproxylic beetle is the first *Cucujus* species known only from Mediterranean and it is probably endemic to Calabria (Italy). The species was found especially in old–growth mountain forests of high conservation value (i.e. national parks) dominated by Calabrian pine (*Pinus laricio calabrica*). We hypothesize that *Cucujus tulliae* sp. n. probably evolved from isolated populations of *Cucujus haematodes* Erichson, 1845. The species is thus relictual and of high conservation value, corresponding at least to endangered (EN) category with respect to recent IUCN criterion. *Cucujus tulliae* sp. n. is here compared with two species native to Europe – *Cucujus haematodes* and *Cucujus cinnaberinus* (Scopoli, 1763) and with the Caucasian *Cucujus haematodes caucasicus* Motschulsky, 1845, which is confirmed as a valid subspecies. The male genitalia of this Caucasian form have been examined and illustrated for the first time. A comprehensive key to adults and larvae of European species is provided.

## Introduction

The genus *Cucujus* F. enumerates at the moment 11 known species, distributed throughout the Holarctic region and highly concentrated in Asia, with many endemics especially in India, Nepal, Myanmar, China, Taiwan and Japan (Horák and Chobot 2009; Lee and Pütz 2008). Only two native species have been known from Europe, *Cucujus cinnaberinus* (Scopoli, 1763), and *Cucujus haematodes* Erichson, 1845. A third species was described as *Cucujus siculus* by Pic (1894) from Sicily; Ratti (1986, 2000) later regarded this taxon as a synonym of the northern American *Cucujus clavipes* (Fabricius 1781), the record being probably based on an accidental introduction into Sicily, or on a mislabeling of the beetle.

*Cucujus cinnaberinus* is an endemic taxon to Europe, distributed from Spain to Ukraine and Sweden, its populations are more densely diffused only in eastern Europe, from Austria and Bavaria eastwards (Horák and Chobot 2009). In Italy the beetle was thought extinct after 1960, but in the last decade it has been found in Piemonte and on the Alburni mountains in the Campania Region by Biscaccianti et al. (2009), and after 49 years absence in the Sila National Park in Calabria (Mazzei et al. 2011).

*Cucujus haematodes* is a palearctic species ranging from Bavaria and Southern Italy to Japan, it comprises at least one well differentiated subspecies, *Cucujus haematodes opacus* Lewis, 1988, found in Japan and Taiwan, whereas the nominal form is known from Southern Italy (Calabria), Greece and Eastern Europe until the Primorskiy Region of far eastern Russia and China (Horák and Chobot 2009). Another form, *haematodes haematodes caucasicus* Motschulsky, 1845, is known from Armenia, Georgia and Russia, but the status of this subspecies was considered more dubious (Horák and Chobot 2009), although Mamaev et al. (1977) in a larval key considered the preimaginal characters as those of a separate species.

During a long term survey of the *Cucujus* populations of the Sila National Park, started 2009 as reported in Mazzei et al. (2011), a large amount of *Cucujus* larvae has been collected and reared until pupation and eclosion. Among large numbers of *Cucujus cinnaberinus* and *Cucujus haematodes* specimens, a third form has been recognized both by larval as well as by adult characteristics. We present here the description of this new *Cucujus* species, that is probably endemic to the Calabrian mountains.

## Material and methods

All the adult specimens have been obtained by rearing the larval material collected in the Sila National Park under the bark of an endemic pine, *Pinus laricio* var. *calabrica* and occasionally of silver fir, *Abies alba* fallen trees. Adult specimens are in our study area quite uneasy to collect in nature, probably because of their early appearance in spring and short reproductive season. On the contrary, the preimaginal stages are well known for their underbark life, in the literature they are sometimes defined as “scavengers”, but also predators on pupae and small larvae of other insects (Mamaev et al. 1977) or even on other subcortical beetles (Rozhkov 1970; Burakowski et al. 1986). Palm (1941) observed that *Cucujus cinnaberinus* larvae are able to feed on bast and cambium, and that cannibalism on younger conspecific larvae ceases in presence of longhorned beetle (Cerambycidae) larvae, that were vigorously accepted. Straka (2008) observed *Cucujus cinnaberinus* adults feeding on various insects and was able to keep larval specimens alive with mealworms – darkling beetle larvae (Tenebrionidae). Horák et al. (2010) observed in far eastern Russia that the associated guild of *Cucujus haematodes* was composed by springtails (Collembola), mites (Acari), ants (Formicidae), carabids (Carabidae) and subcortical histerids (Histeridae). Mazzei et al. (2011) reared *Cucujus cinnaberinus* larvae with calliphorid maggots (Diptera), but especially with fresh beef meat, and this very simple method was adopted in this study.

All the larval specimens were kept at a temperature of 20°C with fresh beef meat, their development lasted about 8 months and a maximum of six larval molts were observed before pupation.

The dissections of adult male specimens were normally performed after short KOH treatment of the terminalia and total abdomen removal. The male genitalia are in fact highly delicate and a less careful extraction may cause the loss of the typical long “flagellum”, well described by Sharp and Muir (1912) and troubles in position of the sclerified structures of the internal sac. Thereafter, the male genital structures were prepared on small transparent labels and enclosed in euparal, finally the labels arranged on the same insect pin of the specimen. The photographs of the adult and larval material were made with a Zeiss Stemi SV11 Stereoscope with a Canon PowerShot G5 five MP digital camera. The male genitalia were photographed with a Zeiss Axioskop equipped with Nomarski optic (Differential Interference Contrast, DIC), using a Nikon Coolpix 4500 four MP digital camera. The male genitalia figures were merged with help of Adobe Photoshop software.

The terminology of adult genitalia follows Wilson (1930) and Thomas (1999), the larval morphology is based on Lawrence (1991).

### Collections examined

Museo Civico di Storia Naturale, Verona, Italy (**MCV**); Department of Entomology the National Museum, Prague, Kunratice 1, Czech Republic (**NMP**); collection P. Brandmayr, conserved in the Department of Ecology of the University of Calabria (**PBC**). The larval material used for the taxonomic key is conserved in 70% alcohol in the “Tullia Zetto” larval collection (**TZC**) in the Department of Ecology of the University of Calabria.

## Results

### Cucujus tulliae sp. n.

urn:lsid:zoobank.org:act:1CC7BB12-8520-4DD0-890F-BDEB5C0FBD82

http://species-id.net/wiki/Cucujus_tulliae

#### Type locality.

Italy, Central Calabria, Sila National Park, mountains between the Cecita Lake and the Longobucco municipality, 1300–1600 m a. s. l., forests between 39°23'/25'N and 16°32'/35'E.

#### Type material.

Holotype male: Sila National Park, Calabria, Italy, Vallone Freddo, Spezzano Sila (CS), 1300 m a. s. l., larva collected at instar five 18.10.2010, lg. Mazzei, adult emerged 28.12.10 in laboratory, prep. N. 3, **PBC**. Paratypes: one female, Vallone Freddo, reared from an aged larva collected 05.04.2011, lg. Mazzei, **PBC**. Larval specimens: 12 larvae (III–VI instar), Calabria, Sila, Vallone Freddo, Spezzano della Sila (CS), 1300 m a. s. l., lg. Mazzei, 05.05.2011; 2 larvae (V–VI), Calabria, Sila, Golia Corvo Natural Reserve, Spezzano della Sila (CS), 1300 m a. s. l., lg. Bonacci; 15.07.2011; 1 larva (V), Calabria, Aspromonte, Gambarie (RC), 1350 m a. s. l., lg. Mazzei, 07.06.2009.

#### Etymology.

Tullia Zetto was an active zoologist and teacher of the Department of Ecology of the University of Calabria, who endeavoured for more than 20 years larval morphology and behaviour of carabids and other predatory beetles in Calabria and in several Mediterranean lands. She was born in Trieste 15.01.1949 and deceased in Cosenza 24.11.2010.

#### Diagnosis.

*Cucujus tulliae* is clearly related to the *cinnaberinus–haematodes* species group, its distribution seems to be restricted to the mountains of Calabrian peninsula. The closest taxon could be *Cucujus haematodes*, from which it can easily be distinguished by the less prominent postgenae, the arrow head shaped prosternal apophysis, the smaller and less spiny pronotum, the typical median lobe of the aedeagus and the larval morphology, that is apparently unique because of its slender body and occipital furrows.

#### Description.

**Figures 1–12.**
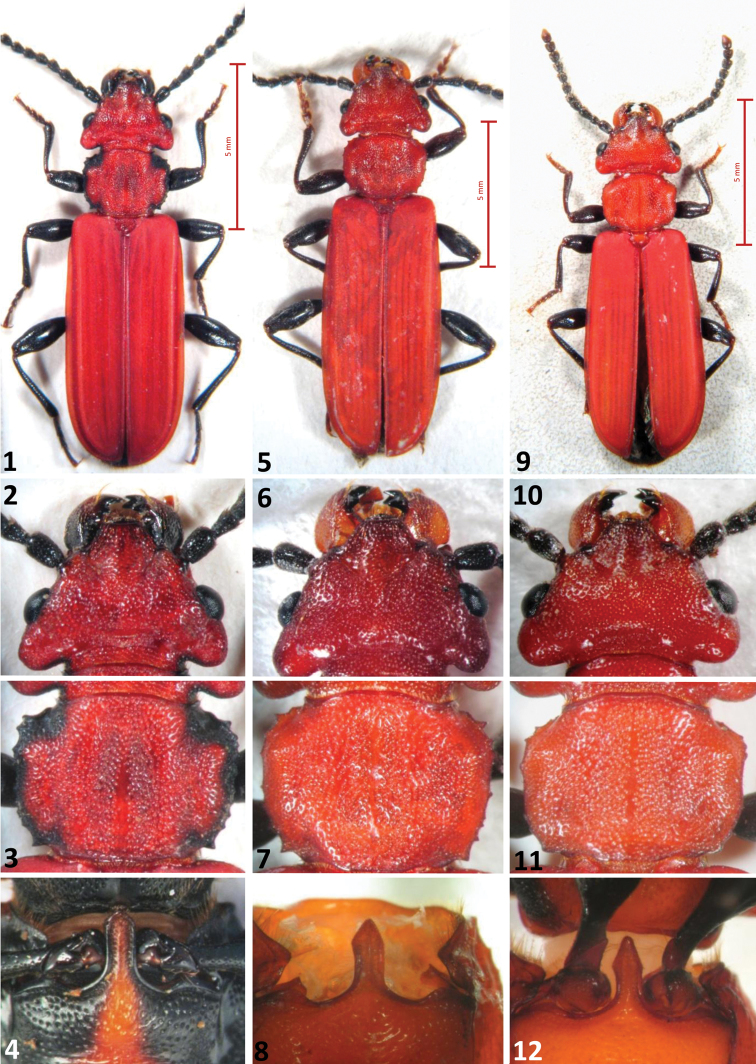
The three European *Cucujus* species, adults. **1–4** *Cucujus cinnaberinus* **1** Total body, dorsal view **2** Head **3** Pronotum **4** Prosternal apophysis **5–8** *Cucujus haematodes* **5** Total body, dorsal view **6** Head **7** Pronotum **8** Prosternal apophysis **9–12** *Cucujus tulliae* **9** Total body, dorsal view **10** Head **11** Pronotum **12** Prosternal apophysis.

**Figures 13–22.**
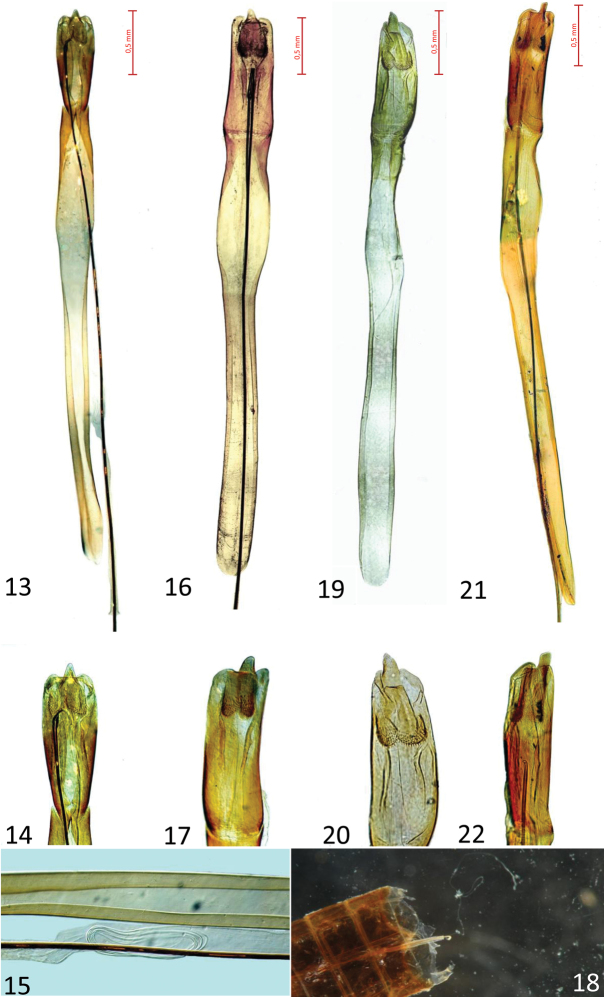
Male genitalia of four *Cucujus* species/subspecies. **13–15** *Cucujus cinnaberinus* (Sila N. Park) **13** Median lobe and median strut with flagellum, dorsal view **14** Median lobe **15** flagellum “ball” inside the endophallus **16–18** *Cucujus haematodes* (Sila N. Park) **16** Median lobe and median strut with flagellum, dorsal view **17** Median lobe **18** Abdominal end of flagellum, with the basal part of the endophallus and genital duct **19–20** *Cucujus tulliae* (Sila N. Park) **19** Median lobe and median strut with flagellum removed **20** Median lobe **21–22** *Cucujus caucasicus* (“Caucasus”) **21** Median lobe and median strut with flagellum, dorsal view **22** Median lobe.

A bright red species, resembling a small–sized *haematodes* in colour, but with a less serrate pronotum. Length 11.2–12.5 mm. Colour light red, legs dark brown/black, tarsi brown. Prosternum of the same colour of pronotum. Antennae black, mandibles red/orange with black apex. Head distinctly wider than pronotum, with two longer setae after the posterior border of eyes. Postgenae less swollen than in *Cucujus haematodes* (Figs 1, 5, 9). Occipital furrow deep and long as one third of the posterior head width. Frontoclypeus with a gentle longitudinal swelling. Pronotum less spiny than in *Cucujus haematodes*, distinctly prolonged at the level of the neck as a short collar (Figs 3, 7, 11). Prosternal apophysis elongated as an arrow head, slender than in *Cucujus haematodes* (Figs 4, 8, 12). Elytral sides distinctly carinate at its upper external margin, almost until the apex. Elytral surface opaque, density of punctures similar to that of *Cucujus haematodes*. Apex of elytra broadly rounded and with short pubescence. Metathoracic wings well developed and robust. Sutural stria slightly convex. Median lobe distinctly wider than median strut, less restricted at its apex than in *haematodes* (Figs 13, 16, 19, 21).Apical process well protruding, more than in *haematodes*, and triangular at the tip, like in *Cucujus cinnaberinus* (Figs 14, 17, 20, 22). Median strut 4.5 times longer than median lobe and particularly thin. Flagellum longer than the entire body, connected at its base with the male deferents, its more chitinized trait reaches the median lobe at the level of the two chitinous plates of the endophallus and bends backwards to the median strut. After bending the flagellum becomes transparent and runs across the endophallus, where, at the middle of its length, it rolls up in a sort of “ball”, like a wire (Fig. 17). In figure 2 the “flagellum ball” is photographed in the median strut of *Cucujus cinnaberinus* (Fig. 18).

#### Distribution and habitat.

The larvae of this species have been collected under the bark of fallen pine trees of at least 25 cm diameter or on dead silver fir fallen trunks, but only on single locations of the National Park. The species seems to prefer cooler, northern exposed slopes and high air humidity at major elevations. In laboratory most larvae kept at 20°C died before pupation, a fact that could be explained by lower temperature preferences of the immature stages. The low number of larvae collected and the successful rearing of only two adults makes hypothesis much more difficult. Concerning geographic distribution, at the moment this taxon is known only from Calabria, in the highest part of the Sila plateau in the core of the Sila National Park, from two sites: Vallone Freddo and Serra Vurga. A single larva has been collected in the surroundings of Gambarie, in the Aspromonte National Park, at 07.06.2009, but this specimen died before pupation. There is little doubt that this new taxon may be an endemic saproxylic beetle of central and southern Calabria, with larval populations depending on the availability of high amounts of dead wood, especially of Calabrian pine, *Pinus laricio* var. *calabrica*.

### Cucujus cinnaberinus

(Scopoli, 1763)

http://species-id.net/wiki/Cucujus_cinnaberinus

#### Note.

We examined several specimens of this taxon, both from Italy as well as from the National Museum of Prague. This species is very abundant in the pine forest of the Sila National Park in Calabria.

#### Examined material.

**PBC**: *Cucujus cinnaberinus*: 1 ♂, Calabria, Sila, Vallone Freddo, Spezzano della Sila (CS), 1300 m a. s. l., lg. Mazzei, 05.04.2011, aedeagus slide n. 2. 1 ♀, Calabria, Sila, Vallone Freddo, Spezzano della Sila (CS), 1300 m a. s. l., lg. Mazzei, 06.05.2011, slide n. 6. 1 ♂, Calabria, Sila, Vallone Freddo, Spezzano della Sila (CS), 1300 m a. s. l., lg. Mazzei, 05.04.2011, slide n. 9. 2 ♂, Calabria, Sila, Monte Pettinascura, San Giovanni in Fiore (CS), 1650 m a. s. l., lg. Mazzei, 19.08.20092 ♀, Calabria, Sila, Monte Pettinascura, San Giovanni in Fiore (CS), 1650 m a. s. l., lg. Mazzei, 29.05.2009. 1♀, Calabria, Sila, Vallone Freddo, Spezzano della Sila (CS), 1300 m a. s. l., lg. Mazzei, 05.04.20113 ♂, Calabria, Sila, Vallone Freddo, Spezzano della Sila (CS), 1300 m a. s. l., lg. Mazzei, 05.04.2011. 1 ♂, Calabria, Sila, Arnocampo, San Giovanni in Fiore (CS), 1250 m a. s. l., lg. Mazzei, 06.07.2009. 1 ♀, Calabria, Sila, Cozzo del Principe, Spezzano della Sila (CS), 1350 m a. s. l., lg. Mazzei, 12.08.2009. 1 ♂, Calabria, Sila, Cozzo del Principe, Spezzano della Sila (CS), 1350 m a. s. l., lg. Mazzei, 12.08.2009. 4 ♂, Calabria, Sila, Golia Corvo Natural Reserve, Spezzano della Sila (CS), 1300 m a. s. l., lg. Mazzei, 07.07.2009. 4♀, Calabria, Sila, Golia Corvo Natural Reserve Spezzano della Sila (CS), 1300 m a. s. l., lg. Mazzei, 07.07.2009.

**MCV**: *Cucujus cinnaberinus*, 1 ♀ ex coll. Brasavola, Sila, Bosco Gariglione, IX. 1 ♂, Calabria, Sila, Silvana Mansio, VIII–1960, det. Ratti, 1971.

**NMP**: about 120 specimens from many countries of Europe.

*Cucujus cinnaberinus* larvae: 5 larvae (V–VI instar), Calabria, Sila, Vallone Freddo, Spezzano della Sila (CS), 1300 m a. s. l., 29.6.2009, lg. Mazzei; 18 larvae (IV–VI), Calabria, Sila, Monte Pettinascura, San Giovanni in Fiore (CS), 1650 m a. s. l., 12.09.2009, lg. Mazzei. 7 larvae (V–VI), Calabria, Sila, Golia Corvo Natural Reserve, Spezzano della Sila (CS), 1300 m a. s. l., 30.05.2009, lg. Mazzei.

### Cucujus haematodes

Erichson, 1845

http://species-id.net/wiki/Cucujus_haematodes

#### Note.

This species is abundant in the Sila National Park, but less than *Cucujus cinnaberinus*. We collected numerous larval stages also in the Aspromonte National Park.

#### Examined material.

**PBC**: *Cucujus haematodes*: 1 ♂, Calabria, Sila, Vallone Freddo, Spezzano della Sila (CS), 1300 m a. s. l., lg. Mazzei, 12.09.2009, slide n. 4. 1♂, Calabria, Sila, Vallone Freddo, Spezzano della Sila (CS), 1300 m a. s. l., lg. Mazzei, 05.04.2011, slide n. 1. 1 ♀, Calabria, Sila, Cozzo del Principe, Spezzano della Sila (CS), 1350 m a. s. l., lg. Bonacci, 12.08.2009, slide n. 7. 1 ♂, Calabria, Sila, Vallone Freddo, Spezzano della Sila (CS), 1300 m a. s. l., lg. Mazzei, 10.12.10, slide n. 8. 1 ♀, Calabria, Sila, Valle di Casu, Longobucco (CS), 1380 m a. s. l., lg. Brandmayr, 11.02.11. 1 ♂ Calabria, Sila, Valle di Casu, Longobucco (CS), 1380 m a. s. l., lg. Brandmayr, 11.02.11. 1 ♂, Calabria, Sila, Monte Pettinascura, San Giovanni in Fiore (CS), 1650 m a. s. l., lg. Bonacci, 29.05.2009. 1 ♀, Calabria, Sila, Cozzo del Principe, Spezzano della Sila (CS), 1350 m a. . l.s, lg. Bonacci, 18.08.2009. 2 ♂, Calabria, Aspromonte, Gambarie (RC), 1350 m a. s. l., lg. Mazzei, 07.06.2009. 3 ♀, Calabria, Aspromonte, Gambarie (RC), 1350 m a. s. l., lg. Mazzei, 07.06.2009. 3 ♂, Calabria, Sila, Colle Vurga, Longobucco (CS), 1550 m a. s. l., lg. Brandmayr, 04.07.2011.

**MCV**: *Cucujus haematodes*: 1 ♀, Calabria, Sila, Bosco Gariglione, IX, coll. ex Brasavola. 1 ♂, Basilicata, Mt. Pollino, Piani del Pollino, VI ’51, lg. Ruffo. 1 ♂, Basilicata, Mt. Pollino, Piano Ruggio, VI–1953, lg. Ruffo.

**NMP**: About 100 specimens from the Czech Republic and to Far East. *Cucujus haematodes* larvae: 3 larvae (V–VI instar), Calabria, Sila, Vallone Freddo, Spezzano della Sila (CS), 1300 m a. s. l., lg. Mazzei, 12.08.2009; 3 larvae (V–VI), Calabria, Sila, Monte Pettinascura, San Giovanni in Fiore (CS), 1650 m a. s. l., lg. Mazzei, 12.08.2009; 1 larva (V instar) Calabria, Sila, Cozzo del Principe, Spezzano della Sila (CS), 1350 m a. s. l., lg. Mazzei, 10.07.2009.

**Figure 23.**
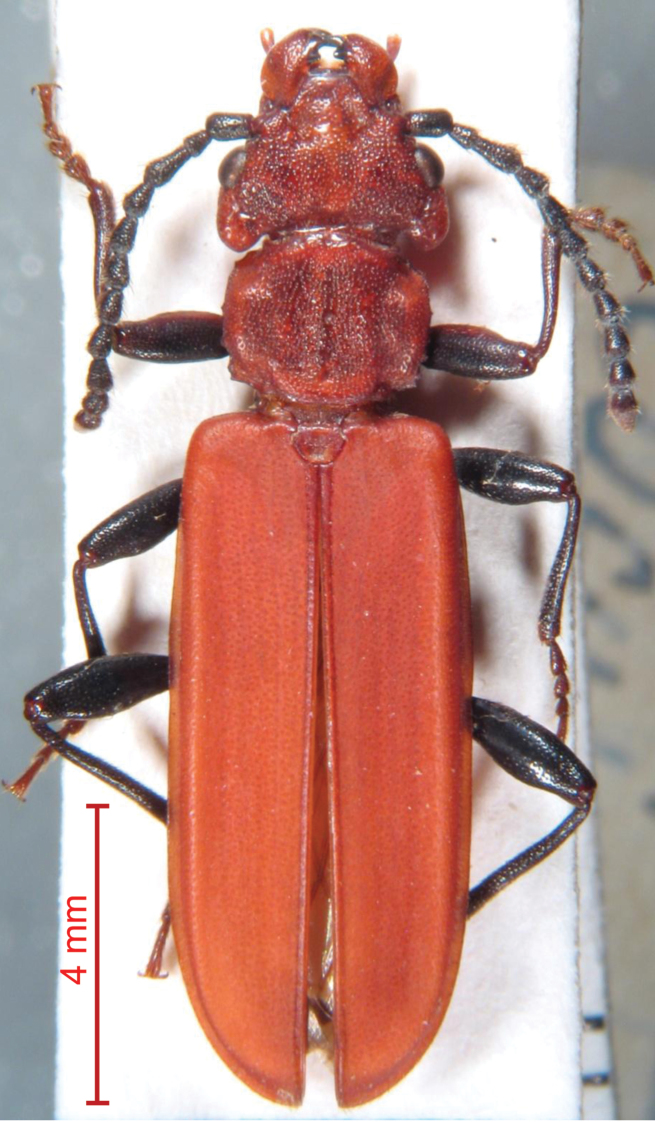
*Cucujus haematodes caucasicus* stat. nov.: dorsal view of male specimen. Locality: Kaukasus, N. W. – Kuban – lg. C. Rost, Berlin.

### Cucujus haematodes caucasicus

Motschulsky, 1845 stat. n.

http://species-id.net/wiki/Cucujus_haematodes_caucasicus

#### Note.

At the beginning of this investigation we faced the possibility that the newly described taxon may belong to another already known form of the *haematodes* group. In Europe only a putative subspecies or relative of *haematodes* was known: *Cucujus caucasicus* Motschulsky, 1845. Horák and Chobot (2009) are of the opinion that: “The state of *Cucujus haematodes caucasicus* is more dubious, however, the main difference from the putative subspecies is the bright red color of the adult mandibles; also, the larval characters correspond with those of a separate species (Mamaev et al. 1977)”. The original description of Motschulsky sounds as follows: “*Beaucup plus grand et plus allongé que le C. depressus, auquel il ressemble pour les couleurs et le fascies. Le corselet est plus transversal. J’ai pris cette belle espèce sur les Alpes du Caucase, sous l’écorce d’un hêtre*”. There is no doubt that the “facies” of *Cucujus caucasicus* was the same of a *Cucujus haematodes*, but larger and with broader pronotum. Also the larval identification key of Mamaev et al. (1977) speaks for a separate species (or subspecies) status. Thus, we examined the male genitalia of three “*caucasicus*” individuals found in the National Museum of Prague.

#### Material examined.

1 ♂ and 1 ♀: Caucasus, “Reitter. Leder.”, determined as: *Cucujus haematodes v. caucasicus* Motsch.”. 1 ♂: Caucasus, “Reitter. Leder.”, determined as: “ *v. caucasicus* – Det. Dr. Obenberger”. 1 ♂: Kaukasus, N. W. – Kuban – C. Rost, Berlin. – ex coll. J. Hlisnikowski. Determined as: “*Cucujus haematodes* Er.”. 1 ♀: Caucasus – Krasnaja Poljana – R. Rous legit. 6.1967. All individuals are conserved in **NMP**.

#### Diagnosis.

A larger *Cucujus haematodes* with a broad pronotum, almost as wide as the head. Median lobe of the same general shape of *Cucujus haematodes*, but the apical process of the inner sac vertical, paddle like in lateral view.

#### Description.

Length 16.0–15.5 mm, colour light red, as in *Cucujus tulliae* sp. n. or in *Cucujus haematodes*, mandibles yellow with brownish/black apex. Head slightly wider than pronotum, postgenae less protrudent sidewards and more rounded (Fig. 23). Prothorax anteriorly not restricted, as it happens in true *Cucujus haematodes*, and proportionally larger, the external borders with pronounced, evident teeth. Prosternal apophysis of the same shape of *Cucujus haematodes*, at the end shortly triangular.

Aedeagus of the same general form of *Cucujus haematodes*, but a little longer, (Figs 21–22), total length mm 5,2 – 5,3, median lobe ending with a vertically flattened apical process, in lateral view distinctly paddle shaped.

#### Distribution and habitat.

This subspecies or allopatric species inhabits the Caucasian mountains – namely Georgia, Armenia and the three Russian regions – Republic of Adigeyia, Krasnodarskiy Kray and Stavropol’skiy Kray (Horák and Chobot 2009).

**Figures 24–35.**
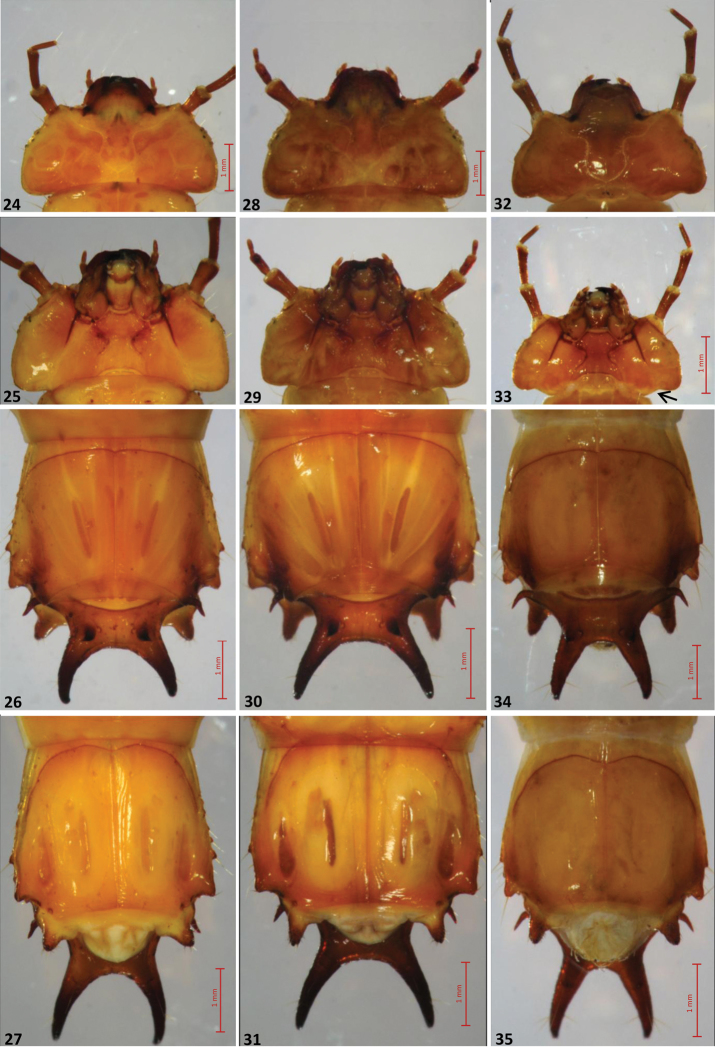
The three European *Cucujus* species, larvae. **24–27** *Cucujus cinnaberinus* **24** Head, dorsal view **25** Head, ventral view **26** Tergum IX and urogomphi, dorsal view **27** Tergum IX and urogomphi, ventral view **28–31** *Cucujus haematodes* **28** Head, dorsal view **29** Head, ventral view **30** Tergum IX and urogomphi, dorsal view **31** Tergum IX and urogomphi, ventral view **32–35** *Cucujus tulliae* **32** Head, dorsal view **33** Head, ventral view **34** Tergum IX and urogomphi, dorsal view **35** Tergum IX and urogomphi, ventral view.

**Figures 36–44.**
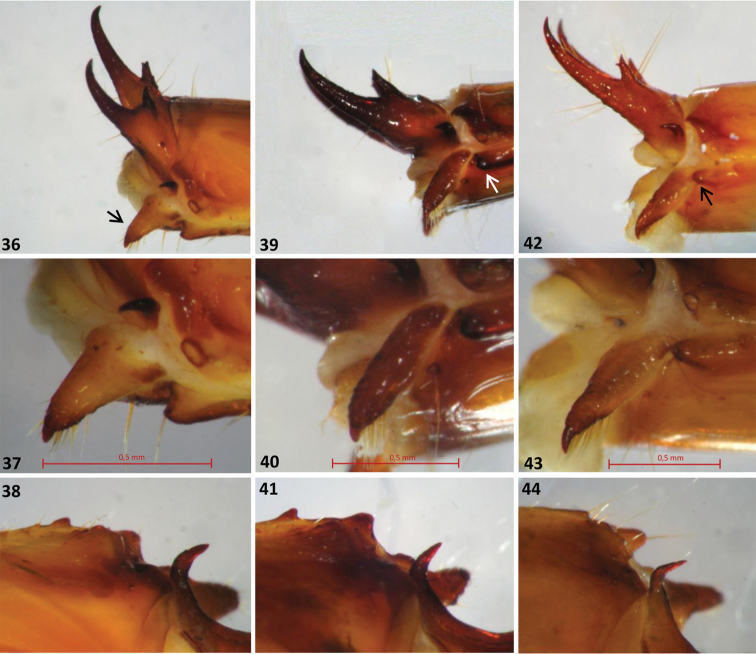
The three European *Cucujus* species, larvae. **36–38** *Cucujus cinnaberinus* **36** Urogomphi, lateral view and conical appendage (black arrow) **36** Conical appendage **38** Lateral end of tergum IX, sclerotized thorn **39–41** *Cucujus haematodes* **39** Urogomphi, lateral view and conical appendage (white arrow) **40** Conical appendage **41** Lateral end of tergum IX, sclerotized thorn **42–44** *Cucujus tulliae* **42** Urogomphi, lateral view and conical appendage (black arrow) **43** Conical appendage **44** Lateral end of tergum IX, sclerotized thorn.

##### Key to the adults of the European species of genus *Cucujus* Fabricius, 1775

**Table:** 

1	Body bright red on the dorsal side, with exception of the apex of the mandibles, that are often lighter, orange. Pronotum entirely reddish, always somewhat restricted at the front border, sides with more or less protruding red teeth	2
–	Sides of pronotum, inner side of postgenae and mandibles black, mandibles before the apex somewhat lighter coloured. Maximum width of the pronotum at the front border, ventral side of the same part black, with a median yellow stripe that prolonges onto the prosternal apophysis. Head triangular, postgenae obliquely protruding backwards, occipital groove well marked and reaching postgenae, nevertheless deeper in the middle, where the punctuation is well developed. Body length 12–15.5 mm, median lobe of the aedeagus evidently restricted at the basis (Fig. 14)	*Cucujus cinnaberinus* Scop.
2	Pronotum robust, with distinct lateral spines, prosternal apophysis with parallel sides and short, triangular end (Fig. 8). Postgenae well developed, body length 13–16 mm	3
–	Pronotum more rounded, lateral borders with small, obtuse spines, on the head side restricted in a short neck. Pronotal apophysis ending with a prolonged, arrowhead like point (Fig. 12). Body length 11.5–12.5 mm	*Cucujus tulliae* sp. n.
3	Pronotum of normal size, distinctly narrower than the head, apical process of the median lobe tongue like, rounded at the tip (Fig. 17)	*Cucujus haematodes* Er.
–	Body size 15.5–16 mm, pronotum broad and robust, little smaller than the head at its posterior end. Postgenae well developed, but less protruding laterally and more backwards oriented, not surpassing the head width at the level of the eyes. Apical process of the median lobe compressed, paddle like (Fig. 22)	*Cucujus haematodes caucasicus* Motsch.

## Larval morphology

### Diagnosis of the genus *Cucujus* Fabricius, 1775

Larvae extremely flat bodied, length 7–8 mm in young stages, around 25–30 mm in aged ones. Colour from pale yellow to dark reddish brown. Head prognathous, always broader than pronotum, maximum width at the occiput (parietale), postgenae inflated. Epicranial suture sinuate, coronal suture short but distinct. Stemmata normally in number of six, located more or less at half of the length of head. Antennae of variable length; first antennomere robust, distal antennomere much slender and setose only at the apex. Second antennomere with a flat sensorium bordered by a chitinous ring. Fronto clypeal suture more less concave in the middle; front margin of the labrum with 4 macrochaetae; frontoclypeal suture absent. Mandibles asymmetrical, broadly triangular and irregular bidentate, the teeth of the left one not at the same level (dorsal view). Two setae on the external side of each mandible; prostheca thin and hook like, broad based. Mola tuberculate, long, anteriorly with a penicillum formed by a dense brush of short setae.

The complex of mouth parts well protruding and occupying more or less one third of the head width. Maxillary and labial palps very short. Legs very short and robust, ending with one tarsungulus.

Notal sclerites distinctly bordered. Abdominal segment VIII longer than precedent, lateral margin with two processes bearing setae. Lateral portion of segment VIII with a stout protrusion below the spiraculum (“spiracular process”). Between segment VIII and IX there is a strong conical appendage with many apical setae. Tergum IX with one pair of strong urogomphi bearing a robust bifid spine at their base. Lateral ends of tergum IX with a strongly sclerotized thorn.

### Key to the larvae of the European species of genus *Cucujus* Fabricius, 1775

**Table:** 

1	Head very wide and flat, posterior margin of the head not excavate, without posterior furrows. Epistomal lateral edge not or less oblique from the antennal basis to the dorsal articulation of mandible. Mouthparts occupant more than a half of the front margin of the head. Antennae not longer than the head. Lateral chitinous thorns of tergum IX gently curved backwards	2
–	Head wide and flat, with distinctly furrowed postgenae at the posterior margin, forming a separate swelling on the occipital part of the head. Mouthparts more protruding, lateral border of epistomal margin distinctly oblique from the antennal basis to the insertion of mandible. Mouthparts complex slender, antennae as long as the whole head. Lateral thorn of the tergum IX strongly bent backwards	3
2	Antennae slender, second joint distinctly longer than the first one, apical joint thin five times longer than wide at the basis. Lateral border of parietale a little swollen in correspondence of the stemmata. Epistomal front margin moderately oblique towards the mandibular basis (Figs 24–25). Urogomphi well curved and apically a little converging to the median plane (Figs 26–27). Basal tooth with minor spine directed outwards and far from the apex. Spiracular process small and little pronounced (Figs 36–38). Conical appendage very large at the basis, distinctly setose around the apical part, chitinous apex short (Fig. 37)	*Cucujus cinnaberinus* (Scop.)
–	Antennae very short, second joint longer like the first one. Apical antennomere four times as wide as at the basis. Head robust and not swollen in the stemmata area (Figs 28–29). Front epistomal margin straight from antennal basis to mandibular insertion. Maximum head width behind the stemmata. Urogomphi well curved but apically not converging to the median plane (Figs 30–31). Basal tooth with less distant apical spines. Conical appendage slender and of different shape (Figs 39–41). Spiracular process inconspicuous	*Cucujus haematodes* Er.
3	Head posteriorly sinuate and marked by very long antennae. Second antennomere a little longer than the first one. Apical antennomere six times longer than wide at the basis. Epistomal front margin strongly oblique towards the mandibular insertion. Frontal suture less sinuate than in previous species (Figs 32–33). Urogomphi strong and a little converging at the end, apex with three evident macrochaetae (Figs 34–35). Basal tooth of normal shape and slender. Spiracular process distinctly protrudent backwards (Figs 42–43). Lateral thorn of tergum IX strongly bent to the rear, forming an angle of ninety degrees (Fig. 44)	*Cucujus tulliae* sp. n.

## Conclusions

The new described taxon of saproxylic beetle, *Cucujus tulliae*, seems to be of limited distribution, at the moment the only two adult specimens, obtained by rearing, are known from Sila National Park, and a single larva has been captured in the Aspromonte National Park, always on dead Calabrian Pine trunks. It is not astounding that this very cryptic species escaped the capture by previous collectors, most of them were occasional visitors of the Sila plateau and this area has never been the object of a long term faunal survey. Moreover, in the older Italian reports (Ratti 1986; 2000) both *Cucujus cinnaberinus* as well as *Cucujus haematodes* were collected mainly on beech (*Fagus sylvatica*) or silver fir and normally in single specimens or very low numbers. The only exception that is known concerns *Cucujus haematodes*, collected by Prof. Sandro Ruffo in more individuals on the Mt. Pollino (Calabria and Basilicata: Italy), in the year 1953, during a kind of “nuptial flight” (personal communication to the last author). Only after 2009 these saproxylic beetles seem to have expanded their habitat also into pure pine woods (Mazzei et al. 2011), perhaps as a consequence of changes in forest management – especially hands–off approach causing higher dead wood accumulations.

The new species seems to be also habitat restricted, in fact the larvae have been found only at three sites in Sila National Park, on northern exposures or at high elevations. Thus, further research is needed to assess the ecological requirements of the species.

The systematic position of *Cucujus tulliae* sp. n. indicates an affinity with *Cucujus haematodes* – body colour and male genital parts, especially the connection between median lobe and median strut (phallobase of other authors) are similar in both species. The new species may have evolved during a cold climate phase (glacial period?) from isolated *Cucujus haematodes* populations. The smaller body size (if confirmed by new findings) speaks for less favorable conditions, as prey density or trunk sizes.

Concerning a first hypothesis on the conservation status of this southern Italian endemic, the evaluation as endangered (EN) in the IUCN categories seems appropriate – e.g. with respect to endemic *Osmoderma* spp. (Audisio et al. 2009). *Cucujus tulliae* should be “ipso facto” included in the Italian red list of saproxylic beetles.

The second outstanding result of this study is that for the first time *Cucujus haematodes caucasicus* Motsch. has been recognized as a valid subspecies, or perhaps as an allopatric species of the *haematodes* “species aggregate”, the heterogeneity of which has been emphasized especially by Horák and Chobot (2009). Also Mamaev et al. (1977), basing on larval characters, considered *Cucujus caucasicus* as a separate species, and a thorough reexamination of V. de Motschoulsky’s diagnosis reveals that later authors severely underestimated his statements.

The *Cucujus haematodes* group reveals to be not only extremely widespread in his Palaearctic distribution, but also highly influenced by isolation and tending to local speciation. A revision of this species aggregate could be of importance for conservation biology and for the relationships between saproxylic predators and history of palearctic forests.

## Supplementary Material

XML Treatment for Cucujus tulliae

XML Treatment for Cucujus cinnaberinus

XML Treatment for Cucujus haematodes

XML Treatment for Cucujus haematodes caucasicus
